# Technical specification for developing a clinical practice guideline for the integration of traditional Chinese medicine and Western medicine

**DOI:** 10.1111/jebm.12654

**Published:** 2024-10-17

**Authors:** Jianxin Wang, Rui Sun, Fengwen Yang, Jianping Liu, Jiajie Yu, Yuanyuan Sun, Xuemin Gao, Boli Zhang, Junhua Zhang, Jinzhou Tian

**Affiliations:** ^1^ Xiyuan Hospital Affiliated to China Academy of Chinese Medical Sciences Beijing China; ^2^ Postdoctoral Research Station of China Academy of Chinese Medical Sciences Beijing China; ^3^ National Medical Products Administration for Laboratory for Clinical Research and Evaluation of Traditional Chinese Medicine Beijing China; ^4^ Beijing University of Chinese Medicine Beijing P. R. China; ^5^ Department of Cardiology Dongzhimen Hospital Beijing University of Chinese Medicine Beijing China; ^6^ Research Center of Traditional Chinese Medicine Tianjin College of Traditional Chinese Medicine Tianjin China; ^7^ Evidence‐Based Medicine Center Tianjin University of Traditional Chinese Medicine Tianjin China; ^8^ Center for Evidence‐Based Chinese Medicine Beijing University of Chinese Medicine Beijing China; ^9^ Chinese Evidence‐Based Medicine Center West China Hospital Sichuan University Chengdu China; ^10^ China Association of Traditional Chinese Medicine Beijing China

**Keywords:** clinical practice guideline, integration, methodological procedure, traditional Chinese medicine, Western medicine

## Abstract

Developing a clinical practice guideline (CPG) for integrating traditional Chinese medicine (TCM) and Western medicine (WM) requires the accurate identification, collation, and integration of all available evidence on TCM and WM in a comprehensive, meaningful, and resource‐efficient manner. This entails framing appropriate clinical questions, retrieving and synthesizing evidence from multiple resources, and providing concise and complete recommendations for specific diseases. However, some existing CPGs for integrating TCM and WM lack deep and organic integration. As the effective preparation of a CPG for integrating TCM and WM typically involves a complex set of principles, methodology, and steps, we believe that a cohesive, step‐by‐step guide on how to prepare a CPG for integrating TCM and WM is essential. To facilitate the design and development of a robust CPG, we present a clear and concise methodology, outlining relevant principles and procedures, supported by references for guidance. This technical specification aims to simplify the methodology for preparing a CPG for integrating TCM and WM; provide healthcare professionals and researchers with methodologically sound tools; and enhance the quality of CPGs for integrating TCM and WM. This technical specification may help elucidate this complex process, facilitate evaluation of the quality of published CPGs for integrating TCM and WM, and improve the understanding and application of recommendations for the combined and integrated use of TCM and WM in a new system.

## 1 INTRODUCTION

China's healthcare policy, dating back to the 1950s, has consistently aimed to support the integration of traditional Chinese medicine (TCM) and Western medicine (WM), resulting in outstanding advancements in public health throughout the country.^1^ According to this policy, equal emphasis on TCM and WM is at the core of the Chinese health service. Central to this endeavor is the development and dissemination of clinical practice guidelines (CPGs) that facilitate the seamless integration of TCM and WM, enabling administrators, practitioners, and patients make informed decisions concerning the appropriate healthcare.^2^ CPGs dedicated to integrating TCM and WM are vital in formulating and applying prevention and treatment strategies, fostering professional development, and achieving higher‐level integration of TCM and WM.^3^

Integration of TCM and WM‐based treatment is practiced in TCM hospitals and general hospitals in China. Integration of TCM and WM, which involves combination of two medical systems, should be applied in disease prevention and treatment by complementing each other and be developed in an integrated manner.^4^, ^5^ However, among the existing WM CPGs in China, only 12% provide recommendations on TCM recommendations.^6^ Similarly, very few WM recommendations were described in TCM CPGs.^6^ Although published CPGs for integrating TCM and WM are becoming increasingly available, the integration of TCM‐ and WM‐based treatment modalities and recommendations is typically described anecdotally, based on expert experience, or is presented simply as the combination of two different sets of treatment options.^7^ Consequently, most of the recommendations in the current CPGs are vague and underspecified. The existing guidelines are deemed unsuitable for clinical practice due to their reliance on expert consensus and insufficient or low‐quality evidence during development. These guidelines often neglect to highlight the strengths of integrating TCM and WM or to clarify the scheduling required when integrating these approaches.^8^ Therefore, higher‐level integrating CPGs, capable of facilitating deep and seamless integration, are required.^9^, ^10^

The technical specification developed here is based on the “Guiding Principles for the Development/Revision of Clinical Diagnosis and Treatment Guidelines in China (2022 Edition)”^11^ and the “Guidance on the Development of Guidelines for Clinical Application of Chinese Patent Drugs in the Treatment of Predominant Diseases.”^12^ This specification provides six fundamental principles and 10 methodological procedures on how to create a CPG to achieve deep and organic integration of TCM and WM.

## 2 FUNDAMENTAL PRINCIPLES OF CPG DEVELOPMENT

### 2.1 Clinical value orientation: Identifying the respective and combined strengths of TCM and WM

The integration of TCM and WM involves more than just merging their practices; rather, in clinical practice, it entails leveraging the strengths of each to address their respective weaknesses.^13^ The key to integrating TCM and WM lies in recognizing these respective strengths and shortcomings in disease prevention, treatment across the disease cycle, and identification of issues that can (or cannot) be resolved through TCM‐WM integration. Accurate positioning is crucial for elucidating the value of integrating TCM and WM in preventing and treating specific diseases or symptoms under specific conditions. Depending on the disease, interventions (which differ depending on the disease stage and type), and desired outcomes (with different treatment objectives leading to different types of outcomes), TCM, WM, or their combination may exhibit different strengths. By effectively integrating these strengths, clinical inquiries can be addressed, clinical needs can be met, and the efficacy of each modality can be demonstrated.^14^

### 2.2 Inclusiveness: Integrating multiple TCM and WM interventions

Few TCM interventions are included in the existing WM guidelines; similarly, few WM interventions are included in the TCM guidelines. In the existing CPGs for integrating TCM and WM, WM recommendations are cited directly or are briefly described, rendering it impossible to produce normative guidance; thus, they are not acceptable to WM practitioners. To address this issue, we propose a strategy where strongly recommended or noncontroversial (or rarely controversial) WM recommendations with specific benefits are directly cited. In contrast, for weakly recommended or highly controversial interventions with unknown benefits, or those lacking explicit recommendations, a systematic review of the evidence should be conducted to update recommendations based on the available evidence. TCM interventions, including commercially available Chinese patent medicines, classic prescriptions, in‐hospital preparations, prescriptions prescribed by renowned and experienced TCM physicians, and TCM techniques (e.g., acupuncture and moxibustion) should be incorporated into the guidelines. However, guideline users are encouraged to select interventions judiciously based on disease specifics, available evidence, and their importance in prevention and treatment.^15^

### 2.3 Multisource evidence from clinical research, ancient TCM literature, and expert empirical evidence

In the last decades, evidence for TCM has emerged from multiple sources and exhibits heterogeneity and diversity. However, traditional evidence‐grading methods, developed within the framework of modern medicine, may not fully accommodate the unique characteristics of TCM in CPGs for integrating TCM and WM. It is imperative to preserve the distinctive features of TCM within these guidelines. Ancient TCM literature and expert experience, which play an unparalleled role in TCM, often do not fit into conventional evidence systems or are undervalued as evidence sources. Ancient TCM literature is a major source of TCM knowledge and experience, while expert experience refers to the valuable experience grained through long‐term clinical practice.^16^

As clinical research evidence of TCM is generally lacking, leveraging the strengths of the ancient TCM literature in a scientifically reasonable, systematic, and normative manner is central to formulating feasible guidelines.^17^ Summarizing, analyzing, and evaluating multiple sources (including ancient TCM literature and expert opinions) and transforming this expertise into evidence‐based recommendations will facilitate the creation of CPGs for integrating TCM and WM.

### 2.4 Integrating prevention and treatment: Emphasizing the concept of “preventive treatment”

“Preventive treatment” is an important concept in TCM and is deeply rooted in classical TCM texts, such as the *Yellow Emperor's Inner Canon*.^18^ This concept emphasizes proactive measures to prevent the occurrence, progression, and recurrence of diseases. In contrast, WM focuses primarily on tertiary prevention of diseases (i.e., “causal prophylaxis, preclinical prophylaxis, and clinical prophylaxis”), corresponding to the concept of “preventive treatment” in TCM. This technical specification emphasizes the integration of TCM and WM in preventing, treating, and rehabilitating and applying TCM in preventing disease occurrence, progression, exacerbation, and recurrence.

### 2.5 Guidelines should be evidence‐based, supplemented by consensus, and refer to expert experience

The development of CPGs for integrating TCM and WM should adhere to the principle of “evidence as a core, consensus as a supplement, and experience as a reference.” To gain acceptance among WM practitioners, TCM recommendations should be grounded in evidence‐based medicine methods recognized internationally. Addressing the research gap in TCM is imperative, particularly by recommending TCM interventions of clinical value even in the absence of robust research evidence. By incorporating TCM clinicians’ expertise, CPGs may effectively integrate evidence‐based WM practices with syndrome‐based differentiation in TCM.

The guidelines for integrating TCM and WM should be developed according to normative standards, employing evidence‐based methodologies, expert consensus, and clinical expertise. These guidelines should capitalize on the best available evidence, identify interventions with significant clinical value, and define integrated recommendations for the combined use of TCM and WM.

### 2.6 Promoting guideline implementation and achieving the organic integration of TCM and WM

Users of CPGs for integrating TCM and WM will encompass TCM and WM practitioners who express interest in TCM and open to its incorporation into their practice.^19^ TCM and WM terms should be mutually translated and integrated. Overcoming language barriers is essential for integrating WM diseases and symptoms with TCM syndromes and formulations, facilitating mutual recognition of concepts and consensus‐building among TCM and WM practitioners regarding recommendations for integrating TCM and WM.

## 3 STEPS AND METHODOLOGY FOR CREATING CPGS FOR INTEGRATING TCM AND WM

### 3.1 Themes and scope

The development of CPGs for integrating of TCM and WM is guided by four key aims: (1) defining an approach for integrating and manifesting the strengths of TCM and WM interventions for each disease; (2) formulating a unified optimal prevention and treatment protocol that does not differentiate between TCM and WM, based on effectiveness, safety, economy, patient acceptability, and comprehensive social effects in disease prevention and treatment; (3) addressing fundamental challenges related to the concurrent and complementary use of TCM and WM; and (4) implementing integrated TCM‐ and WM‐based recommendations.

Several factors affect the scope of these guidelines. First updating WM treatment strategies requires examination of the relationships between WM diseases and TCM diseases, symptoms, and syndromes. Second, the scope of the CPG for integrating TCM and WM should focus on prevention and treatment as key themes. Third, clarification of the optimal timing for applying integrated interventions to maximize their strengths is essential. Finally, methods to achieve integrated TCM and WM‐based prevention and treatment in a focused, objective‐oriented, and principle‐based manner should be described.

The objective of these guidelines is to provide integrated recommendations for prevention and treatment for TCM and WM practitioners, general practitioners, and clinical pharmacists.

### 3.2 Participants

Several groups should be established to oversee the development of the guideline. These include a steering committee, consensus expert panel, clerical panel, working group, and independent review panel. Interdisciplinary collaboration and team cooperation are essential for developing the guidelines. TCM and WM practitioners should participate at a 1:1 ratio, fostering mutual trust, learning, and support.

The establishment of a patient panel is encouraged, enabling patients to contribute to guideline development by expressing their values and preferences regarding clinical questions, recommendation establishment, and guideline dissemination and implementation. All participants involved in guideline development should complete conflict‐of‐interest statements before the official commencement of the process. They should also update their conflict‐of‐interest information promptly during the development process and make their updated information publicly available upon guideline publication.

### 3.3 Protocol and registry

Developing guidelines requires a scientific protocol.^20^ The technical specification proposed here for CPGs integrating TCM and WM, based on the guideline proposal developed by the World Health Organization, underscores two key factors.

First, existing and planned guidelines and expert consensus on target diseases should be systematically screened and cataloged. The relationships between the CPG for integrating TCM and WM and existing TCM and WM guidelines should be defined. Specifically, the recommendations should be consistent, and duplication of work should be avoided to constrain the scope of the guideline and key questions to be addressed. Once objectives are defined, appropriate allocation of research resources and time becomes feasible.

Second, the concepts of “disease and syndrome,” “disease and symptom,” “disease stage and syndrome,” and “multidisciplinary treatment” should be defined in the CPG. To ensure that the guideline is developed in a scientific and transparent manner, its registration with the International Practice Guideline Registry Platform (http://www.guidelines‐registry.cn) is recommended.^21^

### 3.4 Clinical questions and outcomes

#### 3.4.1 Types of clinical questions

The clinical questions included in the CPG for integrating TCM and WM should be designed to assist clinicians in making clinical decisions and should address controversial issues pertinent to physicians and patients. These questions should be structured to delineate the individual and combined strengths of TCM and WM, thereby preventing the squandering of medical resources through their utilization merely as adjunctive treatments or in combined treatment approaches.^22^ The questions should define the target population, predominant disease stage, and primary points of intervention for integrating TCM and WM, elucidating whether, when, and how they should be integrated, as well as the benefits of this integration.

Prevention and treatment, which complement each other, are essential in disease management, as treatment of the present stage represents a form of prevention of the subsequent stage of a disease. Therefore, recommendations for integrating TCM and WM should focus on secondary and tertiary prevention of disease, taking advantage of the strengths of TCM in early detection and treatment to prevent disease progression, aggravation, exacerbation, and recurrence. The implications of cost and resource utilization should be considered during the initial formulation of clinical questions. Clinical experts could collaborate with healthcare economists to incorporate economy‐related questions and establish clinical questions based on cost and resource utilization as the major outcome measures.

#### 3.4.2 Determination of clinical questions

When formulating the clinical questions, emphasis should be given on human‐use experience, the predominant diseases addressed by TCM, unmet clinical requirements, and the disease stage suitable for TCM interventions. This approach will determine the clinical circumstances warranting the integration of TCM and WM.

#### 3.4.3 Importance of clinical questions and outcomes

During the construction of clinical questions, priority should be given to addressing uncertain clinical queries requiring immediate attention. When selecting interventions, the focus should be on those that are known to be efficacious, universally applied, accessible, and well recognized by physicians and patients. Nonprioritized interventions should be screened out to avoid wasting resources, facilitate information gathering via literature review and expert consensus, and increase the clinical feasibility and applicability of the interventions. To ensure that prioritized interventions are appropriately selected, TCM interventions can be selected based on “expert evidence.” Clinical questions pertaining to topics lacking sufficient research evidence can also be prioritized for further investigation to advance clinical research. Table 1 presents a questionnaire for assessing the importance of clinical questions.^23^

**TABLE 1:** Matrix for evaluating the importance of clinical questions.

	Importance evaluation (yes/uncertain/no)^2^					
Proposed clinical questions meriting attention	(1) This question is typically raised in clinical practice, but it has never been fully addressed	(2) This question is difficult to answer accurately	(3) This question has been answered, but new evidence is now available	(4) Measures or methods for addressing this question differ considerably in clinical practice	(5) Addressing this question needs lots of clinical resource and medical cost	Score^[Link]^, ^[Link]^
Clinical question 1						
Clinical question 2						

^1^The importance score is based on the International Guideline Development Credentialing & Certification.

^2^Yes = 1; uncertain = 2; no = 3. The scores for each item are summed to obtain a final score for each question. A lower score indicates lower certain and higher importance of the clinical question.

#### 3.4.4 Transforming clinical questions into scientific research questions

Clinical questions should be systematically transformed into research questions using the Population, Intervention, Comparison, and Outcome (PICO) framework, which facilitates evidence retrieval and appraisal. The quality and utility of clinical questions is undermined if they are incomplete or nonnormatively described. The PICO framework is suitable for transforming questions regarding effective interventions,^24^ and we recommend including all four PICO elements in each question. If a clinical question is expressed as an interrogative sentence, it should address the population (P) and intervention (I), and ideally, either the comparison (C) or outcome (O), or both. In cases where only three PICO elements are included, one should be subdivided, or a “point of comparison” between any two elements should be introduced to ensure completeness.

### 3.5 Evidence acquisition and appraisal

**FIGURE 1:**
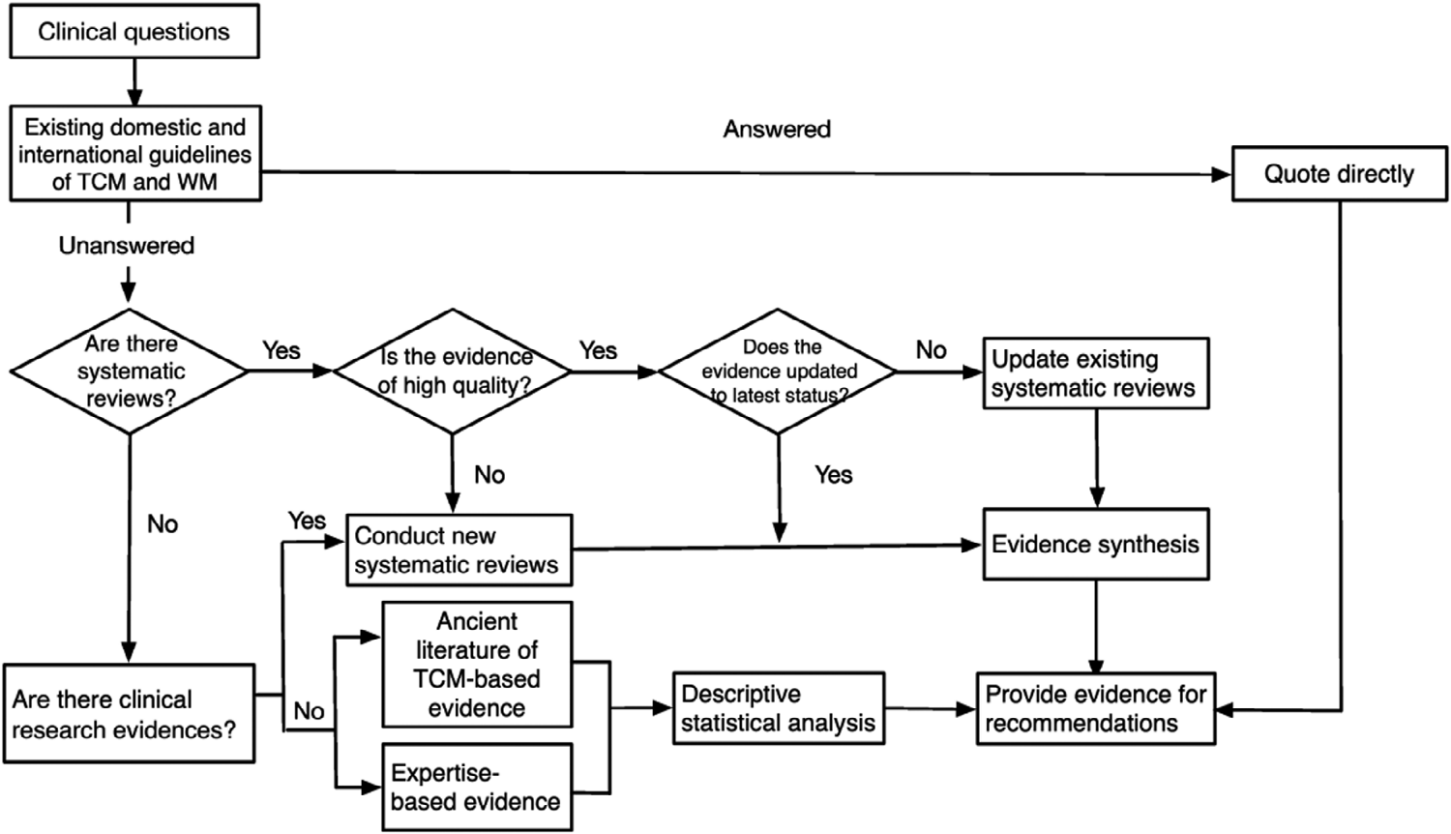
Flow chart of the application of multisource evidence in developing a clinical practice guideline for the integration of Traditional Chinese Medicine and Western medicine.

Evidence acquisition and appraisal are paramount and challenging in guideline development. Guideline developers should formulate evidence‐retrieval strategies based on clinical questions and outcomes. We recommend the systematic incorporation of ancient TCM literature and expert empirical evidence alongside research‐based evidence. Figure 1 illustrates the application of multisource evidence in the development of the CPG for integrating TCM and WM.

#### 3.5.1 Acquisition and appraisal of evidence from existing guidelines

Existing high‐quality evidence‐based guidelines, especially those in WM, can provide the basis for recommendations. Whenever possible, guideline developers should comprehensively evaluate existing guidelines and utilize evidence and recommendations from these high‐quality sources. If the clinical questions align with those in existing guidelines, high‐quality evidence (or strong recommendations based on this evidence) can be directly cited or retrieved and updated. However, for low‐quality evidence, poorly defined evidence, controversial findings, or interventions with weak recommendations, a systematic review should be conducted. Similarly, if the clinical questions do not align well with the existing guidelines, new PICO‐based questions are warranted, and evidence should be re‐retrieved and reviewed systematically

#### 3.5.2 Acquisition and appraisal of existing research evidence

Research evidence forms the cornerstone of guideline recommendations. Classical evidence includes data from systematic reviews and meta‐analyses, while primary research literature encompasses randomized controlled trials, nonrandomized controlled trials, and observational studies.^25^ Real‐world studies (RWSs) have become increasingly important in evaluating the efficacy and safety of TCM. Real‐world evidence derived from RWSs can inform decision‐making in guidelines. To utilize real‐world evidence effectively, it must undergo rigorous and comprehensive appraisal, evaluating both its internal and external validity. The appropriateness, convenience, and accessibility of TCM in real‐world settings should be examined to form a comprehensive evaluation.

#### 3.5.3 Acquisition of nonclinical research evidence

Developing a CPG for integrating TCM and WM is challenging as research evidence is typically lacking. Expert experience in TCM and insights from ancient TCM literature provide unique benefits and play important roles in guideline development. Where evidence is lacking, the following types of evidence should be considered:

1. Ancient TCM literature

The ancient TCM literature is a major source of clinical experience accumulated over thousands of years of practice. In instances where modern research evidence is lacking, guideline developers may find value in consulting this ancient evidence for insights into specific diseases, clinical questions, and approaches to clinical diagnosis and treatment. The selection of ancient literature should be consistent with the clinical questions in the guideline.

The shortcomings of empirical medicine can be effectively remedied by using evidence‐based methods to systematically search, evaluate, screen, and grade the evidence from the ancient TCM literature and by scientifically incorporating patients’ values and clinicians’ experience. The guideline titled “Key technologies and evidence evaluation elements for retrieval of ancient documents of traditional Chinese medicine theory” outlines various sources, elements, terms, and strategies for retrieving ancient literature. This guideline operates under the triple‐combination system of TCM Theory, Human‐Use Experience, and Clinical Trials.^26^

2 Expert‐based empirical evidence

Renowned and highly experienced TCM physicians and experts, with their extensive clinical practice, are acknowledged for their ability to provide effective treatments. Expert experience is critical for rendering TCM medications efficacious. Nonetheless, relying heavily on expert opinion to reach final decisions can easily lead to cognitive bias, reducing the guideline's credibility and transparency. Conversely, if the collection and analysis of expert experience are rigorously designed, standardized, and objective, and if practical and subjective knowledge and skills (expert experience) are transformed into descriptive and objective data and results (expert evidence), the transmission of essential TCM knowledge is significantly enhanced; further, this process contributes valuable evidence that can be used in the development of CPGs. In cases where research evidence is lacking, expert experience can be harnessed and transformed into expert evidence to aid decision‐making in generating recommendations. This approach can bridge the existing gap in TCM research evidence, converting empirical and personal experience into evidential and structural content. It also aids in reducing the degree of heterogeneity in experience and opinions among TCM experts, while increasing the credibility and transparency of the evidence used to develop the recommendations. The standardized collection and presentation of expert evidence can effectively elevate its scientific value and transparency.

##### Economic evaluation

When formulating recommendations for the CPG, it is imperative to consider economic factors to optimize healthcare expenditure and resource allocation, especially regarding the utilization of TCM and WM, either separately or in combination. Economic data include economic evaluation study data and cost‐related research and qualitative data. Economic evaluation studies require retrieval of data from conventional databases and dedicated health‐economics data sources. However, very few economic‐evaluation studies of TCM are publicly accessible, and the available studies often offer low‐quality evidence, which may not meet the standards for guideline development. To address this deficiency, cost‐related qualitative data can be selected, and information on single‐dose costs, defined daily‐dose consumption, course costs, and the price of similar drugs can be gathered via various channels, such as literature review, pricing standards, medical fee schedules, hospital literature, and expert consultation. These data sources provide valuable references for forming recommendations within the guidelines.

#### 3.5.4 Evidence screening and appraisal

Generally, literature retrieval follows the evidence hierarchy pyramid, beginning with the highest quality evidence at the top. Each study's evidence is screened and appraised using the appropriate tools to evaluate the study's methodology.^12^ Appraisal of real‐world evidence is described in the “Technical Specifications for Real‐World Study in TCM: Evidence Quality Evaluation and Reporting.”

### 3.6 Evidence grading system and strength of recommendation

Scientific standards are imperative for grading the quality of evidence and determining the strength of recommendations. Utilizing multiple grading standards is essential to achieve this goal.

The Grading of Recommendations Assessment, Development and Evaluation (GRADE) approach, which is internationally recognized, transparent, and highly applicable, should be employed as the standard for clinical research evidence grading and recommendation formation.^27^

RWSs have been extensively applied, increasing the amount of evidence based on primary research and synthesis. To integrate RWSs effectively, they should be assessed and prioritized according to the GRADE system. The initial quality level of evidence (i.e., the starting point) is high for randomized controlled trials, but lower for observational studies, real‐world single‐arm trials, and nonrandomized trials. Estimating the risk of bias, the first GRADE domain for evaluating evidential quality, can be achieved by evaluating the classic research design (or the corresponding RWS design following modification of the classic research design) using the RWS bias‐evaluation principle. The recommended tool for this task is outlined in the specification document “Technical Specifications for Real‐World Study in TCM: Evidence Quality Evaluation and Reporting.”^28^

Once evidence from the ancient TCM literature and TCM expert sources has been assessed and validated, it can be employed as sources for guidelines. This necessitates the participation of experts in guideline‐development and the full consensus of guideline development panel members.

### 3.7 Formation of recommendations

Recommendations comprise the primary content of the guideline. However, the existing summarized evidence is entirely inadequate for formulating guideline recommendations. To generate the preliminary recommendations required in clinical decision‐making (and to grade their strength), it is important to consider the importance and priority of the questions; the quality and grade of the evidence for TCM interventions; the harm‐benefit analysis (based on the strengths of TCM, such as its complementary or alternative role and clinical efficacy); patient values and preferences; resource use; and the acceptability and feasibility of TCM interventions (Table 2). The evidence‐to‐decision framework (produced by the GRADE Working Group) should be used to develop the recommendations.^29^

**TABLE 2:** Determinants of the strength of recommendations.

Determinant	Description
Importance/priority of the question	The greater the severity and urgency of the question, the more imperative it is to provide a strong recommendation.
Importance of the outcome	The more important the outcome, the stronger the recommendation that is warranted.
Strengths of TCM therapy	When TCM therapy is advantageous in treating a specific disease or a disease stage, can it be used to replace or partially replace WM therapy? What role does TCM play in preventing and treating disease? Can treatment efficacy be improved by using TCM therapy to replace WM therapy or using it as loading therapy?
Benefit‐harm balance (efficacy/harm measurement)	When analyzing the evidence, attention should be directed to the smallest differences in outcomes to assist in judging the benefits and harms. The larger the difference between the benefits and harms, the stronger the rationale for a robust recommendation; conversely, when the disparity is minimal, a weaker recommendation is warranted.
Credibility/quality of evidence	The higher the quality of the evidence, the stronger the recommendation that is warranted.
Values and preferences (patient acceptability)	The larger the difference (or uncertainty) in patient acceptability of the TCM therapy (considering, for instance, convenience of use, compliance with long‐term use, complications, and subjective feelings), the weaker the recommendation that is warranted.
Safety	Will it impose a risk or potential risk on patients? What is the magnitude of the risk?
Economy (resource use)	The higher the costs of treatment (i.e., the more resources it consumes), the weaker the recommendation that is warranted.
Feasibility	Is it included in the national drug reimbursement list or the list of essential medicines? Can operational interventions be performed in a standardized manner? Are additional resources (e.g., training) needed? Are there limitations related to the specific hospital, the geographical location, or legal aspects? The greater the acceptability and feasibility of an intervention, the stronger the recommendation that is warranted.
Equity	The higher the likelihood that an intervention will decrease and enhance equity, the stronger the recommendation that is warranted.

During guideline development, consensus methods, including the Delphi (and modified Delphi) methods, the nominal group technique, and the consensus meeting method, should be flexibly applied based on the specific research contents and objectives. The steps and techniques applied in these consensus methods should be rigorously followed. The guideline should describe, in a standardized and detailed manner, how consensus recommendations were derived, describing the composition of the expert group, the principles used to select experts, the number of participants, the methods used to reach consensus, and the thresholds for reaching consensus. The GRADE grid should be used to reach consensus.

### 3.8 Peer review and consultation on the recommendations

 Peer review and consultation are important for guideline development and quality control. After generating the draft, it is necessary to invite input from all stakeholders through various channels (e.g., at opinion solicitation meetings or by mail). Alternatively, opinions can be solicited via online publication for 1 month. Subsequently, the guideline‐development panel should meticulously collate the received opinions and revise them without deleting or modifying their primary content.

### 3.9 Guideline writing and publication

The development of the CPG should adhere to unified standards outlined in the RIGHT checklist to ensure integrity, transparency, and credibility of the recommendations.^30^

Users of the guideline, particularly WM and general practitioners, may require assistance in accurately understanding the implications and value of integrating TCM and WM for effective implementation of the recommendations. To address this, the developers of the guideline should clearly define the requirements for integrating TCM and WM, outline treatment goals (such as increasing efficacy, improving function or quality of life, and reducing the toxicity of conventional therapy), and specify implementation methods (e.g., complementary, alternative, loading, or escalation therapy). Providing explicit and credible explanations of the recommendations will help clinicians rapidly understand the desirable or undesirable consequences of integrating TCM and WM, thereby enhancing their confidence in these recommendations and motivation to adhere to them.

The CPG should incorporate TCM syndrome differentiation to emphasize the strengths of TCM and clarify its applicability.^31^ To aid guideline users, conventional frameworks and terminology, including recommended models for disease stage, type, symptoms, indicators, and severity, should be integrated into the recommendations. Leveraging clinician experience based on principles of combining disease and syndrome, syndrome and symptoms, and TCM formula and syndrome is essential. Clinical experts should be encouraged to engage in extensive discussions to integrate the WM‐based concept of “disease differentiation” and the TCM‐based concept of “syndrome differentiation.” Detailed interpretations and recommendations should be provided based on clinical practice needs. Therefore, participation of both TCM and WM practitioners in guideline development and interdisciplinary discussions on conceptual framework and terminology is imperative.

### 3.10 Guideline implementation and promotion

Guideline implementation relies on the reliability and practically of the CPG. The successful implementation of a CPG for integrating TCM and WM hinges on national policies and the attitudes toward integration of TCM and WM among clinicians and patients.^32^ Therefore, CPG dissemination and application should be facilitated by interpreting, promoting, disseminating, and implementing it via official websites, the media, academic institutions, and social media; via lectures and lecture tours sponsored by associations or societies; by presenting it in the form of guidelines for general practitioners and patients; and via images and short movies.

Developing a CPG for integrating TCM and WM is a complex procedure. The process involves overcoming numerous challenges stemming from differences in concepts inherent to TCM and WM, cultural disparities, the distinct characteristics of medical systems, and existing national policies. Developing this strategy necessitates progression of TCM and WM opposite directions while concurrently leveraging each other's strengths.

This technical specification scoping review presented here delineates the methods and procedures for developing a CPG for the integration of TCM and WM. It provides recommendations and directions for addressing multiple challenges, such as the nonintegration of TCM‐ and WM‐based organizations and the lack of original methods. Moreover, it underscores the imperative of improving evidence‐handling processes. Finally, it provides greater clarity regarding the integration of TCM and WM, guiding the trajectory of this integration toward more effective and cohesive practices.

## CONFLICT OF INTEREST STATEMENT

Development of this technical specification was initiated by the China Association of Traditional Chinese Medicine, Tianjin University of Traditional Chinese Medicine, and the Chinese Medical Education Association. Xiyuan Hospital (Affiliated to the China Academy of Chinese Medical Sciences), the Center for Evidence‐based Chinese Medicine of Beijing University of Chinese Medicine, and the Chinese Evidence‐Based Medicine Center (West China Hospital), led this development following universal methodology, by reaching consensus based on the latest research evidence and multidisciplinary expert opinion. Every panel member has completed the conflicts of interest disclosure form. No panel member had any financial conflicts of interest. Professional and academic interests were minimized as much as possible. The technical specification will not be disseminated for commercial promotion.

## References

[1] Chen KJ . Evidence‐based clinical Chinese medicine: novel exploration through global vision. Chin J Integr Med. 2019;25:79–80. 10.1007/s11655-019-3459-7 30941680

[2] Jin YH , Wang YP , Xie YL , et al. Research on the development methodology for clinical practice guidelines for organic integration of traditional Chinese and Western medicine. Mil Med Res. 2023;10:45. 37752599 10.1186/s40779-023-00481-9 PMC10523673

[3] Zhang J , Li Y , Zhang B , et al. Evidence‐based traditional Chinese medicine research: Beijing Declaration. J Evid Based Med. 2020;13:91–92. 32470228 10.1111/jebm.12389

[4] Tian G , Zhao C , Zhang X , et al. Evidence‐based traditional Chinese medicine research: two decades of development, its impact, and breakthrough. J Evid Based Med. 2021;14:65–74. 33615709 10.1111/jebm.12420

[5] Robinson N . Integrated traditional Chinese medicine. Complement Ther Clin Pract. 2006;12:132–140. 16648091 10.1016/j.ctcp.2006.01.006

[6] Chen Y , Wang C , Shang H , Yang K , Norris SL . Clinical practice guidelines in China. BMJ. 2018;360:j5158. 29437564 10.1136/bmj.j5158 PMC5797982

[7] Yao S , Wei D , Chen YL , et al. Quality assessment of clinical practice guidelines for integrative medicine in China: a systematic review. Chin J Integr Med. 2017;23:381–385. 27909999 10.1007/s11655-016-2739-z

[8] Tang X , Shi X , Zhao H , et al. Characteristics and quality of clinical practice guidelines addressing acupuncture interventions: a systematic survey of 133 guidelines and 433 acupuncture recommendations. BMJ Open. 2022;12:e058834. 10.1136/bmjopen-2021-058834 PMC8883258 35210347

[9] Zhong LL , Zheng Y , Lau AY , et al. Would integrated Western and traditional Chinese medicine have more benefits for stroke rehabilitation? A systematic review and meta‐analysis. Stroke Vasc Neurol. 2022;7:77–85. 34446530 10.1136/svn-2020-000781 PMC8899656

[10] Wang WJ , Zhang T . Integration of traditional Chinese medicine and Western medicine in the era of precision medicine. J Integr Med. 2017;15:1–7. 28088253 10.1016/S2095-4964(17)60314-5

[11] Yang N , Liu H , Zhao W , et al. Development of the Scientific, Transparent and Applicable Rankings (STAR) tool for clinical practice guidelines. Chin Med J. 2023;136:1430–1438. 37192012 10.1097/CM9.0000000000002713 PMC10278700

[12] Tian J , Zhang B , Gao X , et al. Recommendations for the preparation of clinical application guidelines of the Chinese medicines for the treatment of common diseases. Chin J Integr Med. 2018;38:7–11.

[13] Chen YL , Zhao C , Zhang L , et al. Toward evidence‐based Chinese medicine: status quo, opportunities and challenges. Chin J Integr Med. 2018;24:163–170. 29340887 10.1007/s11655-017-2795-2

[14] Wang Y , Shi X , Li L , Efferth T , Shang D . The impact of artificial intelligence on Traditional Chinese Medicine. Am J Chin Med. 2021;49:1297–1314. 34247564 10.1142/S0192415X21500622

[15] Tang JL , Liu BY , Ma KW . Traditional Chinese medicine. Lancet. 2008;372:1938–1940. 18930523 10.1016/S0140-6736(08)61354-9

[16] Schünemann HJ , Zhang Y , Oxman AD . Distinguishing opinion from evidence in guidelines. BMJ. 2019;366:l4606. 31324659 10.1136/bmj.l4606

[17] Mustafa RA , Garcia CAC , Bhatt M , et al. GRADE notes: how to use GRADE when there is “no” evidence? A case study of the expert evidence approach. J Clin Epidemiol. 2021;137:231–235. 33675954 10.1016/j.jclinepi.2021.02.026

[18] Liu SH , Chen PS , Huang CC , et al. Unlocking the mystery of the therapeutic effects of Chinese medicine on cancer. Front Pharmacol. 2021;11:601785. 33519464 10.3389/fphar.2020.601785 PMC7843369

[19] Peters S , Sukumar K , Blanchard S , et al. Trends in guideline implementation: an updated scoping review. Implement Sci. 2022;17:50. 35870974 10.1186/s13012-022-01223-6 PMC9308215

[20] Chen Y , Guyatt GH , Munn Z , et al. Clinical practice guidelines registry: toward reducing duplication, improving collaboration, and increasing transparency. Ann Intern Med. 2021;174:705–707. 33721516 10.7326/M20-7884

[21] Xun Y , Luo X , Lv M , et al. Protocols for clinical practice guidelines. J Evid Based Med. 2023;16:3–9. 36354129 10.1111/jebm.12502

[22] Fandino W . Formulating a good research question: pearls and pitfalls. Indian J Anaesth. 2019;63:611–616. 31462805 10.4103/ija.IJA_198_19 PMC6691636

[23] Evans L , Rhodes A , Alhazzani W , et al. Surviving sepsis campaign: international guidelines for management of sepsis and septic shock 2021. Intensive Care Med. 2021;47:1181–1247. 34599691 10.1007/s00134-021-06506-y PMC8486643

[24] Cooke A , Smith D , Booth A . Beyond PICO: the SPIDER tool for qualitative evidence synthesis. Qual Health Res. 2012;22:1435–1443. 22829486 10.1177/1049732312452938

[25] Yang J , Ren X , Lv X , et al. Key technologies and evidence evaluation elements for retrieval of ancient documents of “traditional Chinese medicine theory” under the triple‐combination system of TCM theory, human‐use experience, and clinical trials. Chinese Journal of New Drugs. 2023;32:989–993.

[26] Li J , Li B , Zhao XK , Tu JY , Li Y . A critical review to grading systems and recommendations of traditional Chinese medicine guidelines. Health Qual Life Outcomes. 2020;18:174. 32517702 10.1186/s12955-020-01432-x PMC7285562

[27] Technical specifications for real‐world study in TCM: evidence quality evaluation and reporting. J Tradit Chin Med. 2022;63:293–300.

[28] Liu JP . GRADE Methods in traditional medicine. Integr Med Res. 2022;11:100836. 35141135 10.1016/j.imr.2022.100836 PMC8814377

[29] Mercuri M , Baigrie B , Upshur REG . Going from evidence to recommendations: can GRADE get us there? J Eval Clin Pract. 2018;24:1232–1239. 29314554 10.1111/jep.12857

[30] Moberg J , Oxman AD , Rosenbaum S , et al. The GRADE Evidence to Decision (EtD) framework for health system and public health decisions. Health Res Policy Syst. 2018;16:45. 29843743 10.1186/s12961-018-0320-2 PMC5975536

[31] Xie R , Xia Y , Chen Y , et al. The RIGHT extension statement for Traditional Chinese Medicine: development, recommendations, and explanation. Pharmacol Res. 2020;160:105178. 32889127 10.1016/j.phrs.2020.105178 PMC7462769

[32] Jiang M , Lu C , Zhang C , et al. Syndrome differentiation in modern research of traditional Chinese medicine. J Ethnopharmacol. 2012;140:634–642. 22322251 10.1016/j.jep.2012.01.033

